# Hepatitis B virus perceptions and health seeking behaviors among pregnant women in Uganda: implications for prevention and policy

**DOI:** 10.1186/s12913-019-4516-0

**Published:** 2019-10-26

**Authors:** Joan Nankya-Mutyoba, Jim Aizire, Fredrick Makumbi, Ponsiano Ocama, Gregory D. Kirk

**Affiliations:** 1 Department of Epidemiology & Biostatistics, School of Public Health, Makerere University College of Health Sciences, P.O. Box 7072, Kampala, Uganda; 2 Department of Epidemiology, Johns Hopkins Bloomberg School of Public Health, Johns Hopkins University, Baltimore, MD USA; 3 Department of Medicine, School of Medicine, Makerere University College of Health Sciences, Kampala, Uganda; 4 Department of Medicine, School of Medicine, Johns Hopkins University, Baltimore, MD USA

**Keywords:** Hepatitis B, Perceptions, Behavioral intentions, Pregnant women

## Abstract

**Background:**

With most countries in sub-Saharan Africa (SSA) lagging behind schedule to implement a comprehensive viral hepatitis elimination strategy, several barriers to accurate information and hepatitis B virus (HBV) services still exist, that are unique to different regions. In an obstetric population of a high HBV burden SSA setting without antenatal HBV services, we systematically evaluated perceptions and prevention behavioral intentions in relation to HBV and liver cancer.

**Methods:**

Eligible consenting pregnant women were recruited from public health care facilities in the central and northern regions of Uganda, between October 2016 and December 2017. Standardized procedures and instruments based on the health belief model and theory of planned behavior were used to collect data on socio-demographic characteristics, HBV perceptions and behavioral intentions. Descriptive analysis using Chi-square tests was done to obtain distribution of respondents by levels of perceived risk of HBV and liver cancer for themselves, their child under 5 years and their spouse. Modified Poisson regression analyses were used to evaluate relationships between perception variables and different behavioral outcomes (intention to screen, vaccinate and treat HBV).

**Results:**

Perceived risk (PRR = 0.95(0.90–1.00), *p* = 0.055) was inversely associated with intention to screen for HBV. Conversely, perceived self-efficacy showed a consistent association with intention to screen for HBV (PRR = 1.18(1.10–1.23) *p* = 0.005), to vaccinate (PRR = 1.20(1.05–1.36) *p* = 0.006) and to seek treatment for HBV (PRR = 1.40(1.18–1.67) *p* < 0.001). Women from the north, compared to the central region (PRR = 1.76 (1.13–2.72) *p* = 0.012), and those who self-identified as Catholic (PRR = 1.85 (0.99–3.56) *p* = 0.056), and as Protestant, (PRR = 2.22 (1.22–4.04) *p* = 0.002), were more likely to have higher perceived self-efficacy, compared to Muslims. Age and education were not related to perceived self-efficacy.

**Conclusion:**

Women in both regions hold incorrect perceptions of HBV and liver cancer risk, with women from the central reporting higher perceived risk than those from the north. High perceived self-efficacy influenced intention to participate in HBV prevention. Programs and policies geared towards enhancing HBV prevention in this sub-population may consider socio-cultural factors observed to influence prevention behaviors. These findings may guide HBV interventions aimed at improving capacity to seek HBV prevention services, thereby promoting HBV micro-elimination in this sub-population.

## Background

Globally, chronic infection with hepatitis B virus (HBV) is a public health challenge, affecting more than 350 million individuals [1–3]. Chronic HBV infection results in high mortality from cirrhosis and liver cancer [4]. Recent analysis from the global burden of disease data reveals that HBV and its complications of liver cirrhosis and primary liver cancer are not only among the leading 20 causes of death, but are on the rise [5]. The two regions of Africa and south East Asia collectively contribute to the highest HBV prevalence [6] and 70% of liver cancer prevalence worldwide. In accordance with the current global viral hepatitis strategy 2016–2021, elimination goals are to be formulated for identified special sub-populations (micro-elimination), considering both epidemiologic and socio-cultural contexts [7]. Hepatitis B is mostly transmitted horizontally via contact with infected body fluids including blood transfusions and contaminated medical injections, through unprotected sex and from mother to child (vertical transmission) through child birth. In highly endemic regions of SSA, mother to child transmission is recognized as a major route for HBV transmission [8].

With Uganda’s high HBV national prevalence [9], pregnant women need to be considered a focal sub-population for possible viral hepatitis B micro-elimination [7, 10]. Timely antenatal HBV detection, treatment and vaccination to prevent mother to child HBV transmission should result in reduced disease incidence and consequently, prevalence [11]. Yet little has been done to strengthen hepatitis B testing and treatment among Ugandan pregnant women. In most SSA countries including Uganda, specific HBV prevention efforts among pregnant women are hampered by several barriers which include among others, (i) low awareness and knowledge about HBV and its prevention [12, 13] which makes the disease less palpable within communities (ii) health systems that are ill-prepared to offer antenatal screening, treatment and prevention services. Where HBV services exist, they are a private health service, in a few urban healthcare settings and the costs are not affordable. In addition, studies have reported lack of community and peer support as important impediments to HBV prevention [14, 15]. Such structural and financial barriers significantly make uptake of HBV prevention a challenge. (iii) Insufficient information about pregnant women’s, beliefs, perceptions and behavioral intentions in relation to HBV risk and prevention. If inaccurate HBV disease perceptions are not rectified, this negatively affects behavioral response geared towards HBV prevention in this population [16]. Since pregnant women are at risk of transmitting HBV via sexual, vertical and horizontal routes when infected, it remains vital to understand their perceptions of HBV risk, to inform targeted education and risk communication which may enhance HBV prevention behaviors.

Self-perceived health is defined as “an individual’s evaluation of his or her own health” [17]. Theories of health behavior [18, 19] and scientific studies [20–22] have shown that personal perceived threat of disease not only influences one’s personal rating of their health, but also whether they engage in preventive health behaviors. To better understand pregnant women’s HBV and liver cancer-related perceptions, we utilized the health belief model (HBM) [18]. The HBM was selected primarily because of its central attention to disease-preventive health behaviors, and the psychosocial and cognitive determinants of these behaviors, which this study evaluated. The model supposes that individual perceptions of risk of acquiring a given disease and how severe this disease is likely to be, merge to shape overall perceived threat of a given disease. This threat, is further influenced by one’s age, gender and general knowledge about the disease and its causes as individually unique characteristics. It is then weighed against one’s beliefs about the likelihood of receiving care, benefits or barriers to care and ability to seek for and obtain care, to then stimulate care-seeking, treatment and preventive behavior.

### Behavioral intentions

Intention to prevent HBV was hinged on the theory of planned behavior [19], which interprets perceived self-efficacy and individual behavioral control, as predictors of behavioral intention. Behavioral intention, (BI) defined as “*a person’s perceived likelihood or “subjective probability that he or she will engage in a given behavior”* [23], has been shown to be a good proxy measure for actual prevention behaviors in several settings [24–26]*.* The theory of planned behavior has been utilized in disease prevention studies including liver cancer prevention research [27, 28]. Although interventions have been done to elevate population awareness and knowledge of HBV, [29–31] which consequently improves population perceptions about HBV risk and prevention, less work has been done to assess the relationship between HBV perceptions and actual uptake of HBV prevention behaviors particularly in SSA. Continued limited understanding of this relationship may hinder effectiveness of education programs in addressing negative perceptions, which have been identified as barriers to seeking and utilizing prevention services [32]. Applying these two theories of health behavior, we developed and measured constructs for perceptions and behavioral intentions.

In this study, we aimed to measure pregnant women’s perceptions about risk and prevention of HBV and liver cancer; perceived disease severity, barriers, benefits and self-efficacy for hepatitis B and liver cancer, and also determined the relationship between perception variables, socio-demographic characteristics and intention to test, treat and vaccinate against hepatitis B, as proxy measures for actual behaviors.

## Methods

### Study site

This was a cross-sectional study. Participants were recruited from antenatal clinics in public health care facilities. These were considered appropriate settings to access pregnant women across a range of cultural and socio-demographic profiles. Also, antenatal clinic settings would equally be efficient to initiate hepatitis B-specific health education and culturally-suitable prevention messages. Arua hospital was selected in North western region, because it is the main public health facility that offers antenatal care to a large population of the surrounding region and neighboring districts, while in the central region which has a much larger urbanized population, there are many public health facilities that receive high volumes of antenatal clients, therefore two health facilities (Kiswa and Kasangati health centers) were randomly selected from the central region, as previously described [33] .

### Study sample and procedures

A sample size of 455 was estimated using Kish Leslie formula (1965) based on the following assumptions: a proportion who intend to screen for HBV to be 50%, a precision of 0.05, type 1 error (alpha) of 5%, a design effect of 1.2 and a non-response fraction of 10%. Enrollment of expectant women from antenatal clinics was performed each Monday, Tuesday and Thursday of the week in Kiswa and Kasangati health units, and each Monday and Thursday in Arua Hospital, days when the respective antenatal clinics were scheduled to work. Participants were sampled using a systematic sampling approach [34]. Every 5^th^woman waiting in the antenatal clinic line was approached about the study and provided with detailed information, and after completing informed consent procedures was enrolled into the study. This process was conducted until the total sample size was accrued. Women received information about the study on each clinic day from trained study personnel during general antenatal gatherings. Eligible participants had to be pregnant women, at least 18 years of age, residing in the region, able to provide written informed consent and to undergo study procedures. Following information provision, eligible women were approached for details about the study and for permission to participate.

This study sought and obtained ethical clearance. Approval was sought in relation to performing investigation among pregnant women, as a vulnerable group, from Makerere University School of Public health’s Higher Degrees, Research and Ethics Committee.

### Study tool and measures

A structured questionnaire based on the health belief model and the theory of planned behavior was administered to each eligible consenting woman by trained interviewers. To formulate the study instrument, a thorough review of existing literature and of health behavioral theories was conducted. The initial questionnaire was then developed and reviewed by a panel of experts who included 2 hepatologists, a behavioral scientist and a health promotion expert. Their review provided feedback on content and coverage which was used to get a revised version. This was piloted in a sample of 20 pregnant women in a region outside the study area. The pilot provided feedback on language appropriateness and cultural suitability of the questions and from this a final version was obtained and authorized by all experts. The questionnaire had a section on socio-demographic information: age (years) highest education level achieved (none, primary, secondary, vocational and university), region of birth (North, Central, other), current region of residence (North or Central), marital status (single, divorced, monogamously married, polygamously married) and religion (Catholic, Protestant, Muslim, Other). The questionnaire had other sections on knowledge, perceptions and behavioral intentions in relation to HBV, and it has been provided as Additional file 1. The section on perceptions had questions inquiring about individual risk, child’s risk and spouse’s risk of acquiring hepatitis B virus and liver cancer and perceived barriers, benefits and self-efficacy for hepatitis B prevention. The third section had questions assessing behavioral intentions.

#### Perceived risk

Individual perceived risk was assessed using three approaches: i)Absolute lifetime risk (questions: *“What is the likelihood that you will get hepatitis B disease during your lifetime?”* and “*What is the likelihood that you will get liver cancer during your lifetime?”;ii)* conditional risk (questions: “*What is the likelihood that you will get liver cancer during your lifetime, if you were infected with hepatitis B virus?*”) with likert-scale responses *“very low”, “low”, “moderate”, “high and “very high”*). The third approach assessed ‘comparative risk’ (questions: “*What is the likelihood that you will get liver cancer during your lifetime, (a) compared to another woman your age?”(b) “compared to your spouse?”*, with responses of “much higher”, “higher”, “same”, “lower”, “much lower”). To evaluate perceived risk for their child and spouse, participants were asked these questions “*What is the likelihood that your young child (aged 5 years or less) will get (i) hepatitis B (ii) liver cancer during their lifetime?” and* “*What is the likelihood that your spouse will get (i) hepatitis B (ii) liver cancer during their lifetime?”.* Responses were as described for individual risk.

#### Perceived severity, barriers, benefits and self-efficacy

Perceived severity was assessed with these statements [1]; “*If I had liver cancer i)my career would be endangered, ii)my marriage would be endangered, iii) my financial status would deteriorate, iv) I believe that hepatitis B is a serious disease, v)hepatitis B can be fatal and vi) if I got liver cancer, it would more serious than other diseases.”*

Perceived benefits were assessed with these statements; “*If I vaccinate my child against HBV, I do not worry about the child getting liver cancer later in adulthood”, “Testing for HBV will help me find and treat HBV early, before it causes liver cancer”, “If I am tested and found to have HBV, the treatment may not be as bad as treatment for liver cancer” “Testing for HBV is the only way to find out if I have the disease”, “Testing , Immunizing against, and treating HBV is an easy way to prevent liver cancer”, “The HBV test will help you not to worry as much about liver cancer”, “Testing and treating HBV will decrease my chances of dying from liver cancer”*.

Perceived barriers were assessed by these statements; “*Compared with your other health problems, having to test for HBV is not important”, “You are not aware that hepatitis B has a vaccine”, “Adults do not need to test for HBV”, “At your age, you do not vaccinate against HBV”. “You do not need a hepatitis B test or vaccine if you do not have liver symptoms.”, “You are afraid to have a hepatitis B test because it might show that you are infected”, “Having the HBV test is a lot of trouble for you”, “You are worried about having the HBV test because you don’t understand what will be done.”, “Having a hepatitis B test is painful for you.”, “Cost would keep you from having the HBV test.”, “Getting vaccinated for hepatitis B when pregnant will result in a miscarriage*”.

Perceived self-efficacy was assessed with these statements; “*I am certain that I can take my infant for all the recommended immunizations, even if the immunization center is far from where I live”, “I am certain that I can take myself for a hepatitis B test, even if I have to pay for the test”, “I am certain that I can take myself for a hepatitis B vaccination, even if I have to pay for the vaccination”, “If I am tested and found to have hepatitis B, I am certain that I can take myself for hepatitis B treatment, even if the treatment center is far from where I live*”. Responses to perceived severity, benefits, barriers and self-efficacy were “strongly disagree”, “disagree”, “neutral”, “agree”, “strongly agree”, with participants requested to select which of these best represents their belief.

#### Behavioral intentions

Behavioral intentions were assessed by four questions; “*How likely is it that you will take your child for vaccination against HBV, as part of the routine infant immunization schedule?”, “How likely is it that you will take a hepatitis B test over the next 12 months?”, “How likely is it that, if tested and found infected, you will seek treatment for hepatitis B over the next 12 months?”, “How likely is it that, if tested and found unimmunized, you will seek and obtain hepatitis B vaccination over the next 12 months?”*. Responses for this section were on a 5-point likert scale, of “very unlikely”, “unlikely” “neutral”, “likely” and “very likely”, score = 1, if response was “likely” or “very likely”, otherwise score = 0.

### Statistical analysis

Descriptive univariate analysis of socio-demographic and perception variables was performed to obtain distribution for continuous variables and frequencies for categorical variables. The dataset used for this analysis is provided as Additional file 2. Responses to perceived risk of “very low” and “low” were merged, as were responses of “high” and “very high”, to obtain a three-level outcome of “low”, “moderate” and “high” perceived risk. Proportion of participants with low, moderate and high perceived risk for self, for child and for spouse were graphically illustrated, by region. In order to perform regression analyses, all five perception measures (risk, severity, benefits, barriers and self-efficacy) were standardized as follows; scores were generated by summing up responses for each item representing the perception measure. Scores were then standardized by subtracting the mean score from each single score, and dividing the value by the standard deviation of the scores distribution. We used Cronbach’s alpha coefficient to assess internal consistency of scales measuring each perception variable and Spearman’s correlation coefficient to assess correlations between the variables. Cronbach’s alpha coefficients for the scales of perceived risk, severity, barriers, benefits and self-efficacy were 0.569, 0.801, 0.775, 0.823 and 0.786 respectively. Perception variables were entered into the model as continuous variables. We created three behavioral intention variables as outcome variables. Each of the behavioral intention variables (i) intention to test for hepatitis B, (ii) intention to seek hepatitis B treatment (iii) intention to obtain a hepatitis B vaccination, were categorized into 2 categories, with scores of 1 to 3 representing lower intentions and scores of 4 to 5, higher intentions. We then performed a two-step analysis; first, bivariable and multivariable models were fitted using modified Poisson regression to examine relationships between perception variables and each behavioral intention outcome. Factors related to behavioral intention outcomes at bivariable level, with a *p*-value of 0.10 were entered into the multivariable regression model. Interaction terms were also included in the model, and where they were significant, were retained, else they were dropped, for a more parsimonious model. Second, regression analyses were done to further examine the potential relationship between socio-demographic factors and perception variables that were predictive of behavioral intention outcomes. Statistical significance in the multivariable models were determined at the 5% cut-off. Estimated prevalence risk ratios and their corresponding 95% confidence intervals were reported.

## Results

The study involved a total of 455 pregnant women, 300 from the central region and 155 from the north region. Average age (sd) of participants was 24.9 (5.2) years. Just over three quarters (79%) were Christian, and about half (53%) had at least a secondary level education while 34% had primary or no education. Other participant characteristics are shown in Table 1.

**Table 1:** Socio-demographic characteristics of pregnant women who enrolled for the study

Characteristic	Frequency (*N* = 455)	Percent (%)
Age (in completed years)		
≤ 19	65	14.4
20–24	174	38.2
25–29	130	28.6
30–34	58	12.8
≥ 35	26	5.7
Education Level		
None	16	3.6
Primary	140	31.1
Secondary	240	53.3
Post-secondary (vocational)	33	7.3
University	21	4.7
Region of birth		
North	136	30.0
Central	168	37.1
Other	149	32.9
Marital status		
Single/divorced	24	5.3
Monogamy	209	46.4
Polygamy	122	48.7
Religion		
Catholic	157	34.5
Protestant	204	44.8
Islam/other	94	20.7

### Perceptions of risk of acquiring hepatitis B and liver cancer

**Fig. 1:**
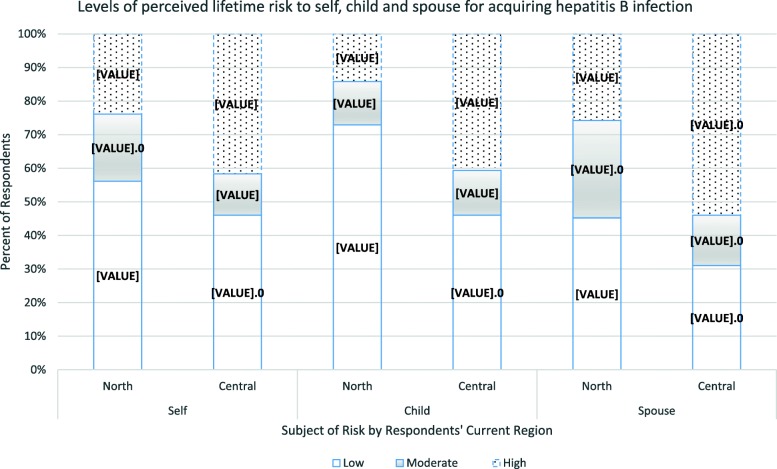
Graph showing perceived lifetime risk for acquiring hepatitis B infection among pregnant women in Northern and Central Uganda. Low = risk was perceived as low, Moderate = risk was perceived as moderate, High = risk was perceived as high. North = participants from the Northern region. Central = participants from the Central region. Self = participants’ perceived risk for themselves. Child = participants’ perceived risk for their child. Spouse = participants’ perceived risk for their spouse

**Fig. 2:**
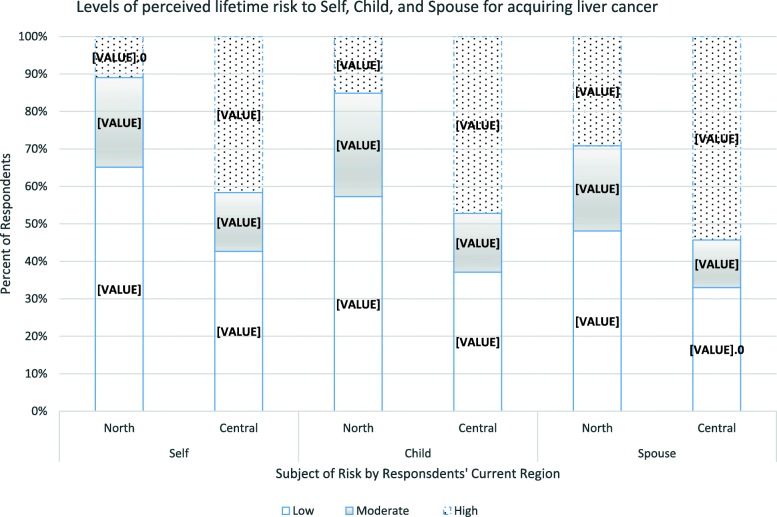
Graph showing perceived lifetime risk for acquiring liver cancer among pregnant women in Northern and Central Uganda. Low = risk was perceived as low, Moderate = risk was perceived as moderate, High = risk was perceived as high. North = participants from the Northern region. Central = participants from the Central region. Self = participants’ perceived risk for themselves. Child = participants’ perceived risk for their child. Spouse = participants’ perceived risk for their spouse

About half of all participants perceived their lifetime risk of acquiring HBV (225/455, 49.5%) and liver cancer (229/455, 50.3%) to be low. More than a third,(161/455, 35.4%), overall, reported low perceived risk of acquiring liver cancer, given HBV infection. There were regional differences in levels of perceived risk of acquiring HBV and liver cancer, by the three measures of risk (absolute, conditional and comparative). As shown in Fig. 1, a larger proportion of women from the north than the central region perceived their individual lifetime risk of getting HBV to be low (56% versus 46%, *p* < 0.01), that of their children to be low (73% versus 46%, *p* < 0.001), and that of their spouses to be low (45% versus 31%, *p* < 0.001). Similarly, as shown in Fig. 2, the northern region had fewer women than the central perceiving their personal lifetime risk of getting liver cancer to be high (11% versus 42%, *p* < 0.001), that of their children to be high (15% versus 47%, *p* < 0.001), and that of their spouses to be high (29% versus 54%, *p* < 0.001).

**Fig. 3:**
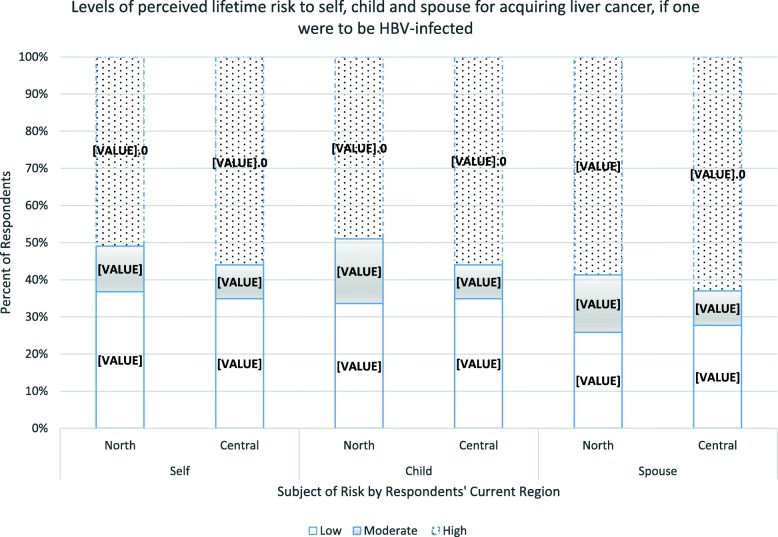
Perception of risk of getting liver cancer for self, spouse and child if one were to be infected with the hepatitis B virus, among pregnant women in Northern and Central Uganda. Low = risk was perceived as low, Moderate = risk was perceived as moderate, High = risk was perceived as high. North = participants from the Northern region. Central = participants from the Central region. Self = participants’ perceived risk for themselves. Child = participants’ perceived risk for their child. Spouse = participants’ perceived risk for their spouse

Regarding perceived risk of acquiring liver cancer, if one were infected with HBV, about half of women from both regions (north 51%, central 56%, *p* = 0.447) perceived themselves to have high risk, while about two thirds (north 59%, central 63% *p* = 0.148) perceived risk to their spouse as high (Fig. 3). Fewer respondents from the north compared to the central region (49%, versus 62% *p* = 0.014) believed that risk to their children getting liver cancer, if infected with HBV was high.

For perceived risk compared to others, approximately a quarter of women in both regions (north 23.4%, central 26.9% *p* = 0.282) perceived their risk of acquiring liver cancer to be higher than that of another woman their age. Regarding women’s perceived risk of getting liver cancer compared to their spouses, a third of women from the central compared to a quarter from the north (31.3% versus 24.5% *p* = 0.003) reported high perceived risk.

### Perception variables and HBV preventive behavioral intentions

The relationships between perceptions variables and intention to screen, vaccinate and treat for hepatitis B were evaluated separately and results shown in three models in Table 2. Models 1, 2 and 3 show unadjusted and adjusted prevalence risk ratios for perceptions and intention to screen for hepatitis B, to seek hepatitis B treatment and to vaccinate against hepatitis B, respectively.

**Table 2:** Poisson regression models of relationships between perception variables and hepatitis B prevention behavioral Intentions

Variable	Unadjusted PRR (95% CI)	*p*-value	Adjusted PRR (95% CI)	*p*-value
Model 1				
Perceived risk	0.98 (0.94–1.00)	0.239	0.95 (0.90–1.00)	0.055
Perceived severity	1.05 (0.99–1.10)	0.085	1.04 (0.97–1.10)	0.312
Perceived benefits	1.04 (0.99–1.10)	0.125	0.97 (0.91–1.03)	0.357
Perceived barriers	**0.97 (0.95–0.99)**	**0.004**	1.00 (0.98–1.01)	0.721
Perceived self-efficacy	**1.18 (1.05–1.31)**	**0.004**	**1.18 (1.05–1.23)**	**0.005**
Model 2				
Perceived risk	0.98 (0.97–1.03)	0.239	0.97 (0.93–1.01)	0.238
Perceived severity	**1.07 (1.01–1.12)**	**0.013**	1.02 (0.96–1.08)	0.577
Perceived benefits	**1.10 (1.04–1.17**)	**0.002**	1.05 (0.97–1.12)	0.237
Perceived barriers	**0.97 (0.95–0.98)**	**< 0.001**	0.99 (0.97–1.00)	0.366
Perceived self-efficacy	**1.38 (1.18–1.62)**	**< 0.001**	**1.40 (1.18–1.67)**	**< 0.001**
Model 3				
Perceived risk	1.00 (0.96–1.03)	0.986	0.92 (0.93–1.03)	0.453
Perceived severity	**1.05 (1.00–1.10)**	**0.040**	1.06 (0.98–1.14)	0.150
Perceived benefits	**1.08 (1.02–1.13**)	**0.010**	1.04 (0.96–1.13)	0.304
Perceived barriers	**0.98 (0.96–0.99)**	**0.018**	1.01 (0.98–1.02)	0.677
Perceived self-efficacy	**1.24 (1.09–1.40)**	**0.001**	**1.20 (1.05–1.36)**	**0.006**

Model 1 shows perceptions variables and intention to screen for hepatitis B. Model 2 shows perceptions variables and intention to seek hepatitis B treatment. Model 3 shows perceptions variables and intention to vaccinate against hepatitis B

#### Intention to screen for hepatitis B

In bivariable regression analyses, perceived barriers and perceived self-efficacy showed an inverse and a direct association with intention to screen for hepatitis B, respectively. For each unit increase in level of perceived barriers to screening, there was a 3% lower prevalence of intention to screen for HBV (PRR = 0.97(0.95–0.99) *p* = 0.004). In multivariable models, perceived self-efficacy showed a consistent association with intention to screen for HBV (PRR = 1.18(1.10–1.23) *p* = 0.005). In addition, there was an inverse association between high perceived HBV risk and intention to screen for HBV (PRR = 0.95(0.90–1.00), *p* = 0.055), after adjusting for other perception variables. See Table 2.

#### Intention to seek for hepatitis B treatment

In bivariate analysis, intention to seek HBV treatment was significantly associated with perceived self-efficacy (PRR = 1.38 (1.18–1.62) *p* < 0.001); perceived disease severity (PRR = 1.07(1.01–1.12) *p* = 0.013) and perceived benefits (PRR = 1.10 (1.04–1.17) *p* = 0.002). There was an inverse association between perceived barriers (PRR = 0.97(0.95–0.98) *p* < 0.001) and intent to seek HBV treatment. However, only perceived self-efficacy persisted in adjusted models for intention to seek HBV treatment ((PRR = 1.40(1.18–1.67) *p* < 0.001).

#### Intention to obtain hepatitis B vaccination

In bivariate analyses, intention to get HBV vaccination was significantly related to perceived self-efficacy (PRR = 1.24 (1.09–1.40) *p* = 0.001); associations with borderline significance were also observed with perceived disease severity PRR = 1.05 (1.00–1.10) *p* = 0.040; and perceived benefits 1.10 (1.02–1.13) *p* = 0.010) and perceived barriers (PRR = 0.98 (0.96–0.99) *p* < 0.018). In the multivariate analysis, only perceived self-efficacy (PRR = 1.20(1.05–1.36) *p* = 0.006) was statistically significantly associated with intent to get HBV vaccination.

#### Socio-demographic factors and perceived self-efficacy

We performed further analysis to determine which socio-demographic variables were related to having high perceived self-efficacy., In a multivariate regression model, respondents from the northern region had 76% higher prevalence of high perceived self-efficacy (PRR = 1.76 (1.13–2.72) *p* = 0.012) compared to those from the central region. In addition, respondents who identified as catholic, (PRR = 1.85 (1.21–3.56) *p* = 0.056) or protestant, (PRR = 2.22 (1.22–4.04) *p* = 0.002) had higher prevalence of perceived self-efficacy compared to those who self-identified as Muslims. As displayed in Table 3, marital status, education and age were not related to having high perceived self-efficacy.

**Table 3:** Multivariable Poisson regression model of socio-demographic factors and perceived self-efficacy

Factor	category	Adjusted PRR (95% CI)	*p*-value
Age group (years)	≤19	1	
	20+	1.09 (0.93–1.27)	0.288
Education	≤Primary	1	
	≥Secondary	0.88 (0.60–1.28)	0.496
Residence	Central	1	
	North	**1.76 (1.13–2.72)**	**0.012**
Religion	Islam	1	
	Catholic	1.85 (0.99–3.56)	0.056
	Protestant	**2.22 (1.22–4.04)**	**0.002**
Marital status	Single/divorced	1	
	Monogamy	0.55 (0.25–1.21)	0.136
	Polygamy	0.64 (0.31–1.32)	0.225

## Discussion

HBV education triggers formulation of decisions to seek HBV care and prevention services, but is more effective if it is rooted in a clear understanding of existing population perceptions regarding disease risks and prevention. In this study, we have assessed perceptions and behavioral intentions related to HBV risk and prevention among pregnant women in two regions of Uganda, a country of moderate to high HBV prevalence with inadequate programs for preventing mother to child HBV transmission. We identified that high perceived self-efficacy was associated with intention to screen, vaccinate and seek treatment for hepatitis B. Further assessment also showed that individuals residing in the north, compared to the central region, plus those belonging to Christian religious following compared to Muslims, were more likely to have high perceived self-efficacy for participating in HBV prevention actions.

Few pregnant women, all-inclusive, had high perceived risk of acquiring HBV and liver cancer during their lifetime, and a significant proportion still believed they were at low risk of liver cancer, even if they were to contract HBV. Our results also showed regional differences in risk perceptions, where a higher fraction of women from the central region tended to have high perceived risk of acquiring HBV and liver cancer, for themselves, their children and their spouses, compared to those from the northern region. This finding is similar to a study by Kue and colleagues among Chinese immigrants in the USA [35], where perceptions about HBV and liver cancer were low in a population at increased HBV risk. There is sufficient evidence linking HBV to liver cancer [36], including evidence that HBV exerts a direct carcinogenic effect on the liver [37–39] and that HBV vaccination [40, 41] and treatment for chronic HBV infection [42] has reduced liver cancer rates in some territories. Moreover, recent findings from SSA showed that liver cancer occurs at a much younger age among HBV-infected individuals [43]. It is therefore important that HBV prevention interventions are based on an understanding of population perceptions regarding HBV and liver cancer risk, to incorporate appropriate risk communication and risk reduction strategies.

Perceived self-efficacy in this study was independently related to hepatitis B prevention behavioral intentions, including intention to screen for hepatitis B, to seek treatment, and to receive a hepatitis B vaccination. In this, our data stand with the theory of planned behaviour, indicating that if individuals are self-assured in their ability to prevent HBV, they are more likely to participate in preventive actions. A recent study among Iranian patients with non-alcoholic fatty liver disease also found that both perceived duration of illness and self-efficacy were predictive of adopting healthy nutritional habits [44]. Similar findings to these have been reported among immigrant minority populations in Europe [45, 46]. However, due to limited number of studies examining this relationship among African populations, more research to uncover other possible factors that explain this relationship would be useful.

Although perceived risk and severity have been reported in several studies to positively influence disease screening behaviors [35, 47–50], and both the health belief model and theory of planned behaviour so stipulate, observations from our study did not support this relationship. Perceived severity was not associated with intention to test, vaccinate or treat HBV, and perceived risk was inversely related to intention to screen for HBV, though the magnitude of association was not strong and was not statistically significant. Pregnant women might care more about consequences of not screening, to their unborn baby, other than to themselves, such that irrespective of the level of perceived risk, they would seek care. The theoretical models therefore might be more applicable to individuals who only consider their own risk, in deciding whether to seek care. In a US study among high risk men, lack of HBV vaccination was found among at-risk men with low perceived risk of HBV [51]. Conversely, another study found perceived severity of HBV disease to be negatively associated with HBV testing [52]. A possible explanation for our findings might be the multiple measures we used to estimate perceived risk, which might have masked the magnitude of risk perception as a construct. Equally, our investigation of risk perceptions and behavioral intentions occurred in a context where HBV services for pregnant women to consider accessing are non-existing [53], whereas in the USA, services are available, at a cost. These differences might influence how individuals perceive risk and how they make decisions on intention to take preventive action.

Links between HBV-related perception constructs and socio-demographic characteristics have been barely evaluated, particularly in SSA pregnant populations, or in developed countries among migrants from Africa. In our study, both region and religion influenced perceived self-efficacy, a socio-cognitive construct shown to positively influence uptake of HBV preventive behaviors. Pregnant women who resided in the central, compared to the northern region were more likely to have low perceived self-efficacy for taking up HBV prevention measures. In addition, individuals who self-identified as Muslims were also more likely to have low self-efficacy for participating in HBV prevention services, compared to those who self-identified as Christians, in adjusted models. These findings mirror those in a study among Moroccan immigrants in Europe [45], where authors reported that influence from Islamic leaders in this minority, mostly Muslim community, negatively influenced intention to participate in HBV screening. It is nonetheless, less clear which factors underlie this finding, and more research might uncover issues not investigated in this study. The finding however, suggests that HBV prevention programs may benefit from being culturally adapted to suit the environments in which they plan to be implemented.

We note that this study had important limitations. Assessment of behavioral intentions relied on participants’ self-reports and HBV-related perceptions were gauged in a setting without a national program for HBV testing or vaccination for pregnant women. This might have influenced how respondents perceived their risk and how they might have reported their intent to participate in HBV prevention services, which may limit comparability to other research. Moreover, we did not include assessment of cues to action. Nonetheless, our study is among very few investigations to evaluate hepatitis B related perceptions and preventive intentions among indigenous African pregnant women. As such, it contributes to filling an existing gap on available evidence to inform programs that aim to reinforce hepatitis B prevention behaviors among pregnant women within the SSA region. In addition, we performed a rigorous assessment of HBV-related perceptions and preventive behavioral intentions, followed by further analysis to specifically identify correlates of perceived self-efficacy in this pregnant population, given that perceived efficacy was directly related to positive HBV preventive behavioral intentions. This work, in line with the current global health sector strategy on viral hepatitis elimination by 2030 [7], has significant implications for national programming and policy. HBV prevention programs should aim to provide accurate risk communication that will enable individuals to correct erroneous perceptions of HBV and liver cancer risk. They should equally purpose to improve self-efficacy in sections of the community with low efficacy, in order to improve uptake of HBV prevention behaviors. Given our findings, national policy may consider transcending traditional prevention approaches to reach out to communities through alternative forums, such as places of worship and leaders of religious groups, for a more sustained and end user-centered response to HBV.

## Conclusion

In an obstetric population of a SSA setting without antenatal HBV services, we systematically evaluated perceptions of hepatitis B and liver cancer risk and attendant benefits, barriers and self-efficacy in relation to prevention behaviors. We found low perceptions of risk of both HBV and liver cancer. We identified that high perceived self-efficacy, of which region and religion were significant determinants, was associated with intention to screen, to vaccinate and to seek treatment for hepatitis B. In environments like this one, where specific evidence needed to refine HBV risk and prevention communication is insufficient, these findings may be relied on to lay a foundation for strengthening HBV and liver cancer risk communication and prevention programming, in order to maximize their impact on national HBV elimination strategies.

## Supplementary information

**Additional file 1.** Knowledge, perceptions and behavioral intentions questionnaire. This is a questionnaire that was developed for this study and used to collect data on HBV knowledge, perceptions and preventive behavioral intentions.

**Additional file 2.** Knowledge, perceptions and behavioral intentions dataset. A dataset provided as excel spread sheets. It contains data on socio-demographic, knowledge, perceptions and behavioral intentions variables that were used to perform analyses for the findings reported in this manuscript. Dataset contains variables pertaining to each respondent: identification number, age, gender, nationality, religion (age, gender, nationality and religion of respondent), primary tribe (respondent’s ethnic tribe), marital status (form of marital union), education level (highest education attained), variables c1-c6 describe participant’s personal risk of getting HBV or liver cancer, c7-c13 describe participant’s perceived risk of their child under five years getting HBV or liver cancer, c14-c18 describe participant’s perceived risk of their spouse getting HBV or liver cancer. c1ds-c8ds describe perceived HBV disease severity, pm1-pm7 describe perceived benefits, pb1-pb11 describe perceived barriers, pse12-pse15 describe perceived self-efficacy, bi01-bi04 describe behavioral intentions.

## Data Availability

Both the questionnaire used for data collection, and the dataset containing variables that were analysed to obtain findings that formed the basis of write of this manuscript have been availed as additional files.
