# Correction to: Hepatitis B virus perceptions and health seeking behaviors among pregnant women in Uganda: implications for prevention and policy

**DOI:** 10.1186/s12913-019-4767-9

**Published:** 2019-12-20

**Authors:** Joan Nankya-Mutyoba, Jim Aizire, Fredrick Makumbi, Ponsiano Ocama, Gregory D. Kirk

**Affiliations:** 1 Department of Epidemiology & Biostatistics, School of Public Health, Makerere University College of Health Sciences, P.O. Box 7072, Kampala, Uganda; 2 Department of Epidemiology, Johns Hopkins Bloomberg School of Public Health, Johns Hopkins University, Baltimore, MD USA; 3 Department of Medicine, School of Medicine, Makerere University College of Health Sciences, Kampala, Uganda; 4 Department of Medicine, School of Medicine, Johns Hopkins University, Baltimore, MD USA

**Correction to: BMC Health Serv Res (2009) 19:760**

**https://doi.org/10.1186/s12913-019-4516-0**

In the original publication of this article [1], some values are missing in the Figure 1, Figure 2 and Figure 3. These errors were introduced during typesetting; thus the publisher apologizes for this error. Additionally, the original manuscript has also been updated to amend this error. The correct figures are shown below:

**Fig. 1:**
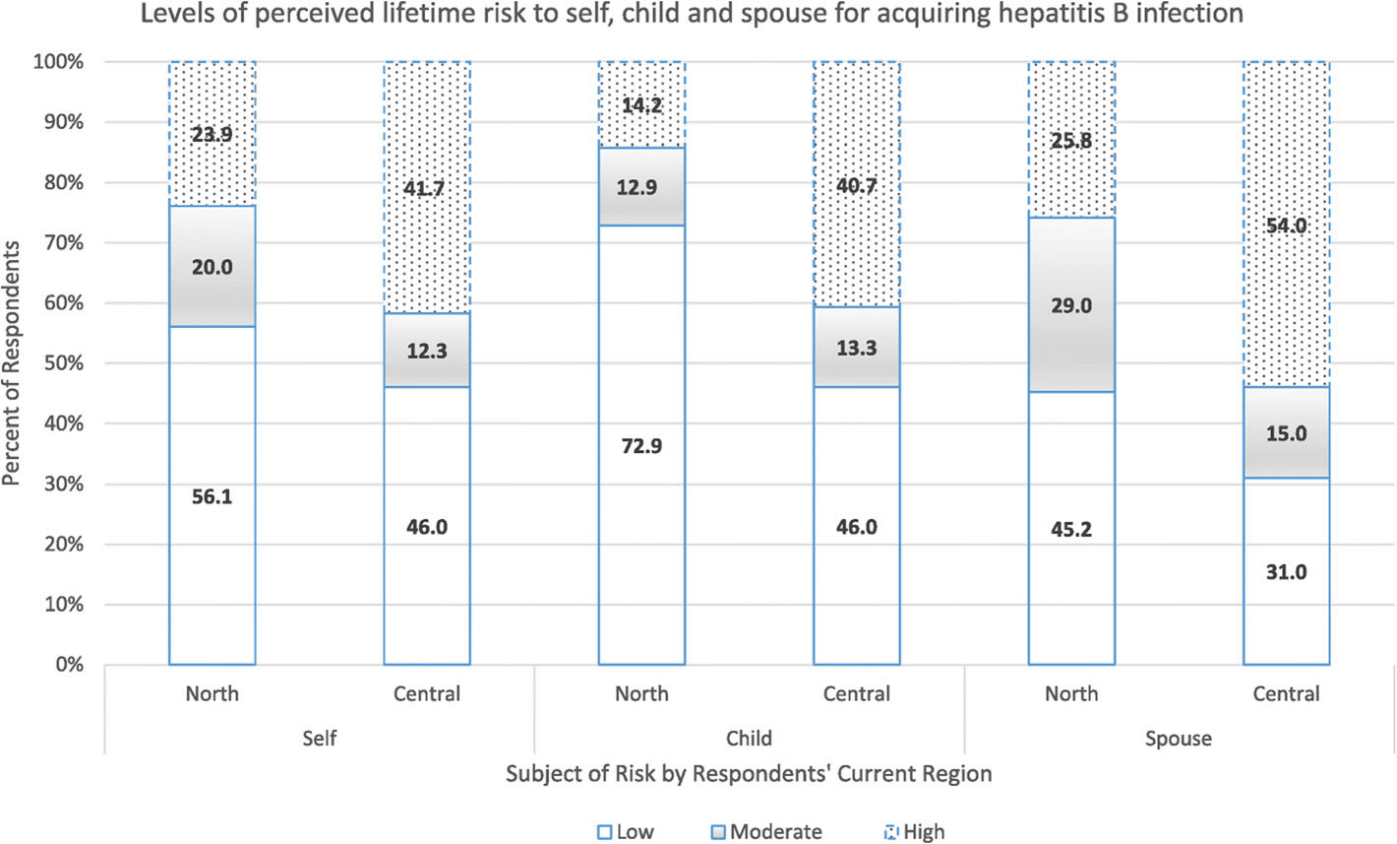
Graph showing perceived lifetime risk for acquiring hepatitis B infection among pregnant women in Northern and Central Uganda. Low = risk was perceived as low, Moderate = risk was perceived as moderate, High = risk was perceived as high. North = participants from the Northern region. Central = participants from the Central region. Self = participants’ perceived risk for themselves. Child = participants’ perceived risk for their child. Spouse = participants’ perceived risk for their spouse

**Fig. 2:**
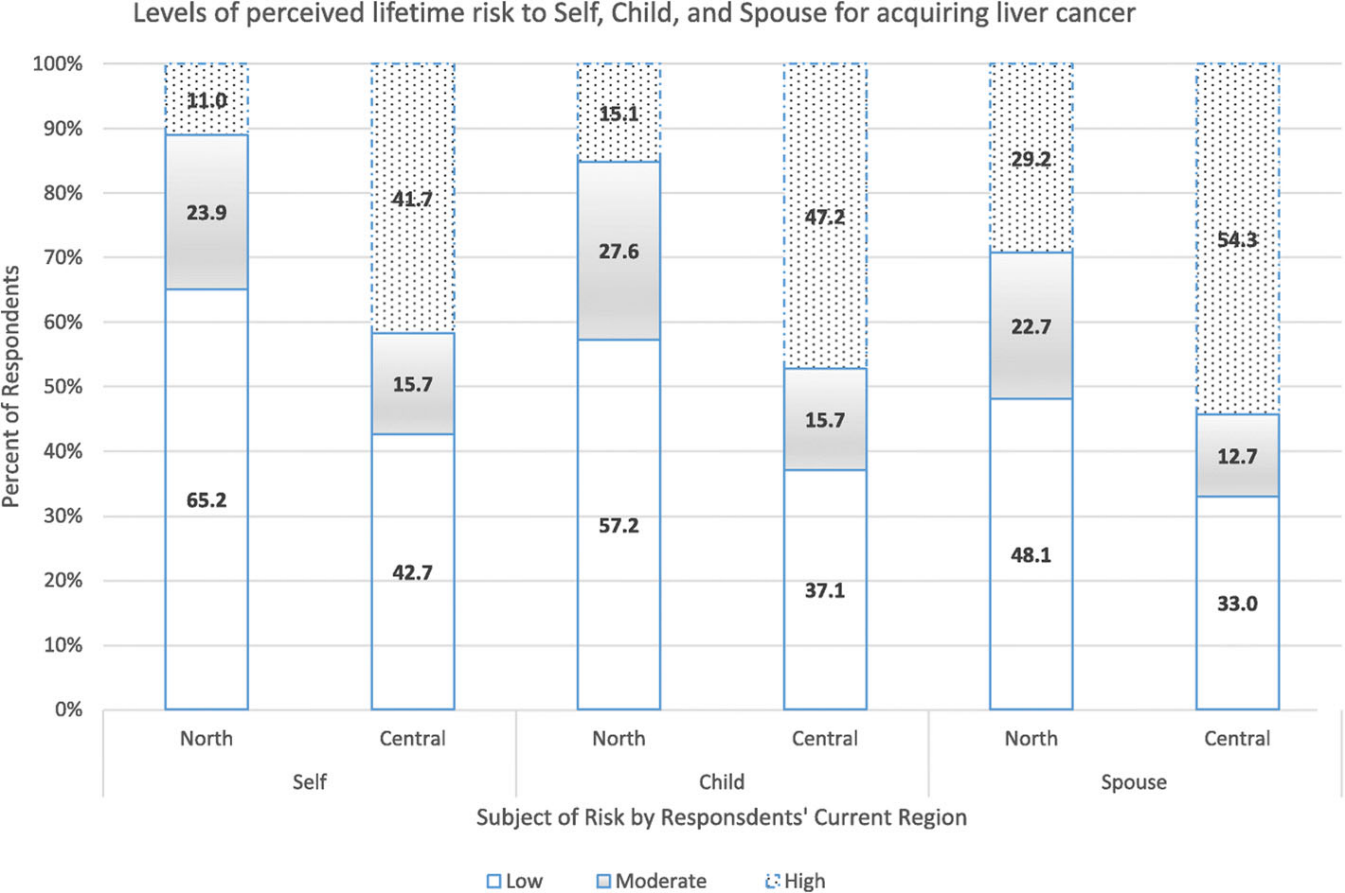
Graph showing perceived lifetime risk for acquiring liver cancer among pregnant women in Northern and Central Uganda. Low = risk was perceived as low, Moderate = risk was perceived as moderate, High = risk was perceived as high. North = participants from the Northern region. Central = participants from the Central region. Self = participants’ perceived risk for themselves. Child = participants’ perceived risk for their child. Spouse = participants’ perceived risk for their spouse

**Fig. 3:**
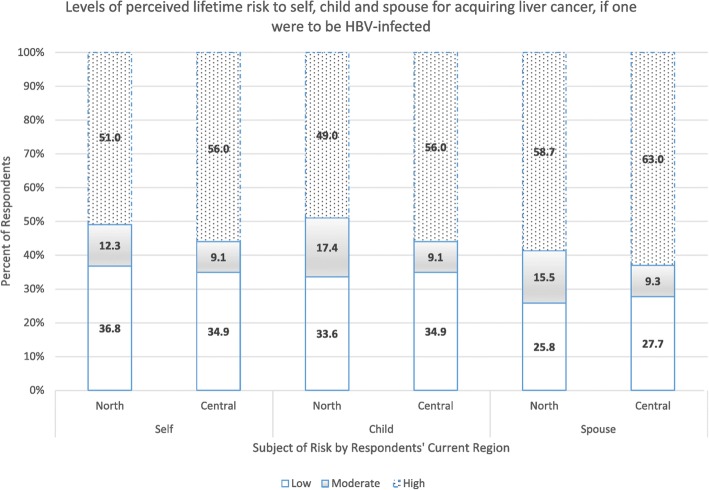
Perception of risk of getting liver cancer for self, spouse and child if one were to be infected with the hepatitis B virus, among pregnant women in Northern and Central Uganda. Low = risk was perceived as low, Moderate = risk was perceived as moderate, High = risk was perceived as high. North = participants from the Northern region. Central = participants from the Central region. Self = participants’ perceived risk for themselves. Child = participants’ perceived risk for their child. Spouse = participants’ perceived risk for their spouse

.
