# Supplementary material for: Hepatitis B virus perceptions and health seeking behaviors among pregnant women in Uganda: implications for prevention and policy
Source: BMC Health Serv Res. 2019 Oct 26;19:760. doi: 10.1186/s12913-019-4516-0 (PMC6815411; doi:10.1186/s12913-019-4516-0)
Supplement: Supplementary file 1 — Additional file 1. Knowledge, perceptions and behavioral intentions questionnaire. This is a questionnaire that was developed for this study and used to collect data on HBV knowledge, perceptions and preventive behavioral intentions. [file 12913_2019_4516_MOESM1_ESM.docx]

## HBV KNOWLEDGE, PERCEPTIONS & BEHAVIORAL INTENTIONS QUESTIONNAIRE

PARTICIPANT ID: **H2U- HA**-⬜⬜⬜ STUDY CODE: H2U

**A. DEMOGRAPHIC INFORMATION**

1. Birth Date: DD ⬜⬜ MM ⬜⬜ YYYY ⬜⬜⬜⬜ **OR**  Age ⬜⬜

2. Gender: 1. ⬜ Male 0. ⬜ Female

3. What is your nationality? (*Check all that apply*)
 ⬜ Ugandan ⬜ Sudanese

⬜ Kenyan ⬜ Rwandan

⬜ Tanzanian ⬜ Congolese

⬜ Other nationality, specify: _____________________

4. What is your region of birth? (*Check only one*)

 ⬜ Central Uganda ⬜ Western Uganda ⬜ Not in Uganda

⬜ Northern Uganda ⬜ West Nile

⬜ Eastern Uganda ⬜ Other Uganda, specify:_____________________

5. What is your primary tribe? (*Use code list below to record tribe affiliation*) ⬜⬜

6. What is your religion? (*Check all that apply*)

⬜ Roman Catholic ⬜ Anglican ⬜ Jehovah’s Witness

⬜ Muslim ⬜ 7^th^ Day Adventist ⬜ Traditional

⬜ Born Again/Evangelical/Pentecostal ⬜ Other:_______________

7. What is your current marital status?

⬜ Single (Never Married/Not Cohabitating) ⬜ Cohabitating

⬜ Married (Mono) ⬜ Married (Poly)

⬜ Divorced or separated ⬜ Widowed

8. What is your highest level of education?

⬜ None or Kindergarten ⬜ Institution (Professional School)

⬜ Primary school ⬜ University

⬜ Secondary school

**B:** **KNOWLEDGE ABOUT HBV and LIVER CANCER**

31. Now you are going to be asked what you know about two diseases, namely 1) hepatitis B and 2) liver cancer. Please tell us what you know, to the best of your ability. You are also going to be asked about what you believe or think regarding these two diseases, and what actions, if any, you plan to do which may affect the way in which people may acquire or protect themselves from these diseases.

| **Awareness and knowledge of hepatitis B /Liver cancer:** | **Yes** | **No** | **Don’t**  **know** |  |  |
| --- | --- | --- | --- | --- | --- |
| 1. Are you aware of a disease called hepatitis B? |  |  |  |  |  |
| 1. Can you mention which part of the body is affected by hepatitis B? (*tick ‘yes’, if correct body part, symptom, etc is mentioned*) |  |  |  |  |  |
| 1. Can you mention at least one symptom or sign of hepatitis B? |  |  |  |  |  |
| 1. Can you mention one way in which the germs that cause hepatitis are transmitted? |  |  |  |  |  |
| 1. Can you mention one disease that hepatitis B causes? |  |  |  |  |  |
| 1. Compared to HIV, the virus that cause AIDS, is hepatitis B less infectious (**01**), similar in infectiousness (**02**) or more infectious (**03**)? | ⬜ **01** ⬜ **02** ⬜ **03** | | | | ⬜ **03** |
| *For statements below, respond with TRUE, FALSE or don’t know* | **True** | **False** | **Don’t**  **k now** |  |  |
| 1. Transmission of hepatitis B can occur through needle sharing |  |  |  |  |  |
| 1. Transmission of hepatitis B can occur sexually |  |  |  |  |  |
| 1. Transmission of hepatitis B can occur from a mother to a child during pregnancy |  |  |  |  |  |
| 1. Transmission of hepatitis B can occur through body contact from adults to children within a household |  |  |  |  |  |
|  |  |  |  |  |  |
| 1. Transmission of hepatitis B can occur through a mosquito bite |  |  |  |  |  |
| 1. Transmission of hepatitis B can occur through sharing cups or cooking utensils |  |  |  |  |  |
| 1. Transmission of hepatitis B can occur through practices such as pre-chewing or oral warming of children’s food |  |  |  |  |  |
| 1. Transmission of hepatitis B can occur through a curse or witchcraft |  |  |  |  |  |
| 1. Hepatitis B virus can cause liver cancer |  |  |  |  |  |
| 1. Men are more likely to acquire liver cancer than women |  |  |  |  |  |
| 1. Hepatitis B can be prevented through vaccination |  |  |  |  |  |
| 1. Liver cancer can be prevented through vaccination |  |  |  |  |  |

**C. PERCEPTIONS ABOUT HEPATITIS B and LIVER CANCER: MEASURES**

**Perceived risk**

**Participant’s personal perceived risk**

1. What is the likelihood that you will get hepatitis B disease during your lifetime?

Very Low ⬜ Low ⬜ Moderate ⬜ High ⬜ Very High ⬜

1. What is the likelihood that you will get liver cancer during your lifetime?

Very Low ⬜ Low ⬜ Moderate ⬜ High ⬜ Very High ⬜

1. What is the likelihood that you will get liver cancer during your lifetime, if you are infected with hepatitis B?

Very Low ⬜ Low ⬜ Moderate ⬜ High ⬜ Very High ⬜

1. What is the likelihood that you will get liver cancer during your lifetime, if you are not infected with hepatitis B?

Very Low ⬜ Low ⬜ Moderate ⬜ High ⬜ Very High ⬜

1. What is the likelihood that you will get liver cancer during your lifetime, compared to another woman your age?

Much higher ⬜ Higher ⬜ Same ⬜ Lower ⬜ Much Lower ⬜

1. What is the likelihood that you will get liver cancer during your lifetime, compared to your spouse?

Much higher ⬜ Higher ⬜ Same ⬜ Lower ⬜ Much Lower ⬜

**Participant’s perceived risk for their child**

1. What is the likelihood that your young child (aged 5 years or less) will get hepatitis B disease during their lifetime?

Very Low ⬜ Low ⬜ Moderate ⬜ High ⬜ Very High ⬜

1. What is the likelihood that your young child (aged 5 years or less) will get hepatitis B disease during their lifetime **if vaccinated** against it?

Very Low ⬜ Low ⬜ Moderate ⬜ High ⬜ Very High ⬜

1. What is the likelihood that your young child (aged 5 years or less) will get hepatitis B disease during their lifetime **if not vaccinated** against it?

Very Low ⬜ Low ⬜ Moderate ⬜ High ⬜ Very High ⬜

1. What is the likelihood that your young child will get liver cancer during their lifetime?

Very Low ⬜ Low ⬜ Moderate ⬜ High ⬜ Very High ⬜

1. What is the likelihood that your young child will get liver cancer during their lifetime, if he/she gets hepatitis B?

Very Low ⬜ Low ⬜ Moderate ⬜ High ⬜ Very High ⬜

1. What is the likelihood that your young child will get liver cancer during their lifetime, if he/she gets vaccinated against hepatitis B?

Very Low ⬜ Low ⬜ Moderate ⬜ High ⬜ Very High ⬜

1. What is the likelihood that your young child will get liver cancer during their lifetime, if he/she does not get vaccinated against hepatitis B?

Very Low ⬜ Low ⬜ Moderate ⬜ High ⬜ Very High ⬜

**Participant’s perceived risks for their spouse**

1. What is the likelihood that your spouse will get hepatitis B disease during their lifetime?

Very Low ⬜ Low ⬜ Moderate ⬜ High ⬜ Very High ⬜

1. What is the likelihood that your spouse will get liver cancer disease during their lifetime?

Very Low ⬜ Low ⬜ Moderate ⬜ High ⬜ Very High ⬜

1. What is the likelihood that your spouse will get liver cancer during their lifetime, if he gets hepatitis B?

Very Low ⬜ Low ⬜ Moderate ⬜ High ⬜ Very High ⬜

1. What is the likelihood that your spouse will get liver cancer during their lifetime, if he gets vaccinated against hepatitis B?

Very Low ⬜ Low ⬜ Moderate ⬜ High ⬜ Very High ⬜

1. What is the likelihood that your spouse will get liver cancer during their lifetime, compared to another man his age?

Much higher ⬜ Higher ⬜ Same ⬜ Lower ⬜ Much Lower ⬜

**PERCIEVED DISEASE SEVERITY**

| **Perceived hepatitis B /Liver cancer disease severity: measures** | | | | | | | | | | | |
| --- | --- | --- | --- | --- | --- | --- | --- | --- | --- | --- | --- |
| **Statement (severity measures )** | | **Response scale** | | | | | | | | | |
| Response (Strongly agree(**SA**), agree(**A**), neutral (**N**), Disagree,(**D**) strongly disagree (**SD**) | | **SA** | | **A** | | | **N** | | **D** | | **SD** |
|  | | **1** | | **2** | | | **3** | | **4** | | **5** |
| 1. If I had liver cancer my career would be endangered. | |  | |  | | |  | |  | |  |
| 1. I believe that hepatitis B is a serious disease | |  | |  | | |  | |  | |  |
| 1. Liver cancer would endanger my marriage | |  | |  | | |  | |  | |  |
| 1. My feelings about myself would change if I got liver cancer. | |  | |  | | |  | |  | |  |
| 1. My financial security would be endangered if I got liver cancer | |  | |  | | |  | |  | |  |
| 1. Problems I would experience from liver cancer would last a long time | |  | |  | | |  | |  | |  |
| 1. If I got liver cancer, it would be more serious than other diseases | |  | |  | | |  | |  | |  |
| 1. If I had liver cancer, my whole life would change. | |  | |  | | |  | |  | |  |
| **Perceived benefits: measures** | | | | | | | | | | | |
| Response (Strongly agree(**SA**), agree(**A**), neutral (**N**), Disagree,(**D**) strongly disagree (**SD**) | **SA** | | **A** | | | **N** | | **D** | | **SD** | |
|  |  | |  | | |  | |  | |  | |
| 1. If I vaccinate my child against HBV, I do not worry about the child getting liver cancer later in adulthood |  | |  | | |  | |  | |  | |
| 1. Testing for HBV will help me find and treat HBV early, before it causes liver cancer |  | |  | | |  | |  | |  | |
| 1. If I am tested and found to have HBV, the treatment may not be as bad as treatment for liver cancer |  | |  | | |  | |  | |  | |
| 1. Testing for HBV is the only way to find out if I have the disease |  | |  | | |  | |  | |  | |
| 1. Testing , Immunizing against, and treating HBV is an easy way to prevent liver cancer |  | |  | | |  | |  | |  | |
| 1. The HBV test will help you not to worry as much about liver cancer |  | |  | | |  | |  | |  | |
| 1. Testing and treating HBV will decrease my chances of dying from liver cancer |  | |  | | |  | |  | |  | |
| **Perceived Barriers: measures** | **Response scale** | | | | | | | | | | |
| Response (Strongly agree(**SA**), agree(**A**), neutral (**N**), Disagree,(**D**) strongly disagree (**SD**) | **SA** | | **A** | | **Neutral** | | | **D** | | **SD** | |
| **Response score** | **5** | | **4** | | **3** | | | **2** | | **1** | |
| 1. Compared with your other health problems, having to test for HBV is **not** important |  | |  | |  | | |  | |  | |
| 1. You are not aware that hepatitis B has a vaccine |  | |  | |  | | |  | |  | |
| 1. Adults do not need to test for HBV |  | |  | |  | | |  | |  | |
| 1. At your age, you do not vaccinate against HBV |  | |  | |  | | |  | |  | |
| 1. You **do not** need a hepatitis B test or vaccine if you do not have liver symptoms. |  | |  | |  | | |  | |  | |
| 1. You are afraid to have a hepatitis B test because it might show that you are infected |  | |  | |  | | |  | |  | |
| 1. Having the HBV test is a lot of trouble for you. |  | |  | |  | | |  | |  | |
| 1. You are worried about having the HBV test because you don't understand what will be done. |  | |  | |  | | |  | |  | |
| 1. Having a hepatitis B test is painful for you. |  | |  | |  | | |  | |  | |
| 1. Cost would keep you from having the HBV test. |  | |  | |  | | |  | |  | |
| 1. Getting vaccinated for hepatitis B when pregnant will result in a miscarriage |  | |  | |  | | |  | |  | |
| **Perceived hepatitis B /liver cancer prevention self-efficacy** | | | | | | | | | | | |
| Response (Strongly agree(**SA**), agree(**A**), neutral (**N**), Disagree,(**D**) strongly disagree (**SD**) | **SA** | | **A** | | **Neutral** | | | **D** | | **SD** | |
| **Response scale** | **1** | | **2** | | **3** | | | **4** | | **5** | |
| 1. I am certain that I can take my infant for all the recommended immunizations, even if the immunization center is far from where I live |  | |  | |  | | |  | |  | |
| 1. I am certain that I can take myself for a hepatitis B test, even if I have to pay for the test |  | |  | |  | | |  | |  | |
| 1. I am certain that I can take myself for a hepatitis B vaccination, even if I have to pay for the vaccination |  | |  | |  | | |  | |  | |
| 1. If I am tested and found to have hepatitis B, I am certain that I can take myself for hepatitis B treatment, even if the treatment center is far from where I live |  | |  | |  | | |  | |  | |

**D. BEHAVIORAL INTENTIONS (BI): MEASURES**

| **Behavioral intentions related to hepatitis B /Liver cancer prevention: measures** | | | | | |
| --- | --- | --- | --- | --- | --- |
| **Question** | **Response scale** | | | | |
| **Response:** Very likely (VL) Likely (L) Neutral (N)  Unlikely (UL) Very Unlikely (VUL**)** | **VL** | **L** | **N** | **UL** | **VUL** |
|  | **1** | **2** | **3** | **4** | **5** |
| 1. How likely is it that you will take your child for vaccination against HBV, as part of the routine infant immunization schedule? |  |  |  |  |  |
| 1. How likely is it that you will take a hepatitis B test over the next 12 months? |  |  |  |  |  |
| 1. How likely is it that, if tested and found infected, you will seek treatment for hepatitis B over the next 12 months? |  |  |  |  |  |
| 1. How likely is it that, if tested and found unimmunized, you will seek and obtain hepatitis B vaccination over the next 12 months? |  |  |  |  |  |
|  | | | | | |

*Thank you for your participation*
